# Monitoring of Gene Expression in Bacteria during Infections Using an Adaptable Set of Bioluminescent, Fluorescent and Colorigenic Fusion Vectors

**DOI:** 10.1371/journal.pone.0020425

**Published:** 2011-06-03

**Authors:** Frank Uliczka, Fabio Pisano, Annika Kochut, Wiebke Opitz, Katharina Herbst, Tatjana Stolz, Petra Dersch

**Affiliations:** 1 Department of Molecular Infection Biology, Helmholtz Centre for Infection Research, Braunschweig, Lower Saxony, Germany; 2 Department of Microbiology, Technical University Braunschweig, Braunschweig, Lower Saxony, Germany; University of Louisville, United States of America

## Abstract

A family of versatile promoter-probe plasmids for gene expression analysis was developed based on a modular expression plasmid system (pZ). The vectors contain different replicons with exchangeable antibiotic cassettes to allow compatibility and expression analysis on a low-, midi- and high-copy number basis. Suicide vector variants also permit chromosomal integration of the reporter fusion and stable vector derivatives can be used for *in vivo* or *in situ* expression studies under non-selective conditions. Transcriptional and translational fusions to the reporter genes *gfp_mut3.1_*, *amCyan*, *dsRed2*, *luxCDABE*, *phoA* or *lacZ* can be constructed, and presence of identical multiple cloning sites in the vector system facilitates the interchange of promoters or reporter genes between the plasmids of the series. The promoter of the constitutively expressed *gapA* gene of *Escherichia coli* was included to obtain fluorescent and bioluminescent expression constructs. A combination of the plasmids allows simultaneous detection and gene expression analysis in individual bacteria, e.g. in bacterial communities or during mouse infections. To test our vector system, we analyzed and quantified expression of *Yersinia pseudotuberculosis* virulence genes under laboratory conditions, in association with cells and during the infection process.

## Introduction

Expression of fluorescent and bioluminescent proteins in bacteria has greatly enhanced our ability to study the molecular mechanisms implicated in pathogen-host cell interactions and allowed us to follow the course of infections in animal models. In particular autofluorescent proteins, such as the monomeric green fluorescent protein (GFP) from the jellyfish *Aequorea victoria*, have been widely used for monitoring gene expression and visualizing intracellular bacteria and proteins [1], [2], [3]. GFP contains an intrinsic chromophore surrounded by a cylinder formed by eleven antiparallel strands, which requires oxygen for cyclization [4], [5]. Up to date many GFP derivatives and other identified autofluorescent proteins are in use, which exhibit a higher fluorescence intensity, improved photostability and distinct excitation or emission spectra [6], [7], [8]. This allows simultaneous detection of multiple fluorescent proteins in a single cell required for co-localization studies and comparative gene expression analysis. Most commonly used autofluorescent proteins are GFP derivatives with enhanced green (EGFP), blue (BFP), cyan (CFP) or yellow (YFP) fluorescence, as well as the tetrameric red fluorescent protein (RFP) from reef corals of the genus *Discosoma* and its improved variants mCherry and DsRed2 [9], [10], [11], [12]. They can be detected under a large variety of different *in vitro* and *in vivo* conditions with UV-light, by fluorometers and fluorescence-activated cell sorting (FACS), and certain combinations of the fluorescent proteins allow multi-labelling experiments.

Besides autofluorescent proteins, luciferases have become very useful for real-time, low-light-imaging of gene expression in individual cells, cell cultures, whole organisms and transgenic animals. Luciferases are enzymes that emit light in the presence of oxygen and a substrate (luciferin). The bacterial luciferin is a reduced riboflavin phosphate (FMNH_2_) that is oxidized by a luciferase in association with a long-chain aldehyde and an oxygen molecule. The bacterial derived *luxCDABE* operon encodes the luciferase (LuxA, LuxB) and the enzymes that produce its substrate (LuxC, LuxD, LuxE). Cells expressing the operon emit light at 490 nm spontaneously, which can be easily detected with high resolution by a photon-counting CCD camera [13]. Luciferase imaging allows detection and monitoring of gene expression in real-time in living single and multi-cellular organisms. Furthermore, it permits analysis of spatial, cell-type, tissue- and host-specific localization of bacteria during infection in genetically modified organisms [13], [14].

Depending on the purpose, it may also be desirable to use *lacZ*, *phoA*, *gusA* or *cat* as colorigenic reporter genes, which have been originally used for promoter identification and targeted gene expression analyses in various organisms [15], [16], [17]. They are very easy to quantify, insensitive to certain environmental factors (e.g. pH, oxygen, light) and have an increased sensitivity with low background reactivity compared to the fluorescent reporters. In addition, many hypersensitive detection systems, antibodies and applicable test strains are available for their analysis.

Due to the frequent use of the different reporter proteins, an extensive number of reporter gene fusion and expression vectors are already available [13], [18], [19]. However, we considered, it would be a great advantage if a variety of colorigenic, bioluminescent and fluorescent proteins were available in a compatible plasmid set with identical multiple cloning sites, in which the origin of replication and the antibiotic resistance cassette are easily interchangeable. This would allow simultaneous expression analysis on a single, low-, midi- and high-copy number basis in different strain backgrounds, harboring different antibiotic resistance cassettes. Furthermore, promoters of interest could easily be switched between the vectors for detection and/or appropriate quantification of weakly and highly expressed genes encoding proteins or non-coding RNAs. Expression of the reporters on stable vector derivatives and suicide vector variants which allow integration of the reporter fusions into the genome can be used for long-term infection studies. In addition, promoters of constitutively expressed genes can be used to obtain fluorescent and bioluminescent strains for *in vivo* detection and imaging.

## Materials and Methods

### Ethics Statement

All animal work was performed in strict accordance with the German regulations of the Society for Laboratory Animal Science (GV-SOLAS) and the European Health Law of the Federation of Laboratory Animal Science Associations (FELASA). The protocol was approved by the Niedersächsisches Landesamt für Verbraucherschutz und Lebensmittelsicherheit: animal licensing committee permission no. 33.9.42502-04-055/09. All efforts were made to minimize suffering.

### Bacterial strains and growth conditions

The bacterial strains and plasmids used in this study are listed in Figure **S1**. *Escherichia coli* and *Salmonella enterica* serovar Typhimurium strains were grown at 37°C in Luria–Bertani broth (LB). *Yersinia pseudotuberculosis* was grown at 25°C in LB. Antibiotics were used at the following concentrations: chloramphenicol 30 mg/ml; kanamycin 40 µg/ml, tetracycline 5 µg/ml and carbenicillin 50 µg/ml.

### DNA manipulation for vector constructions

Standard DNA manipulations, plasmid DNA isolation and transformation in *E. coli* were performed as previously described [20]. All enzymes were purchased from New England Biolabs (Ipswich, USA). Chromosomal DNA of *E. coli* DH10beta was isolated by the UltraClean Soil DNA Isolation Kit (MoBio, Germany). Synthetic oligonucleotides and sequencing primers were supplied by Metabion (München, Germany). Amplified and cloned DNA inserts of the vectors were confirmed by sequencing (GATC, Germany). Plasmids and primers used for DNA sequencing and vector construction in this study are listed in Figures **S1**, **S2**. The nucleotide sequences of the vectors pFU31-pFU99 listed in Figures **S1** were submitted to the NCBI Genbank with accession numbers JF796078–JF796100.

**Figure 1:**
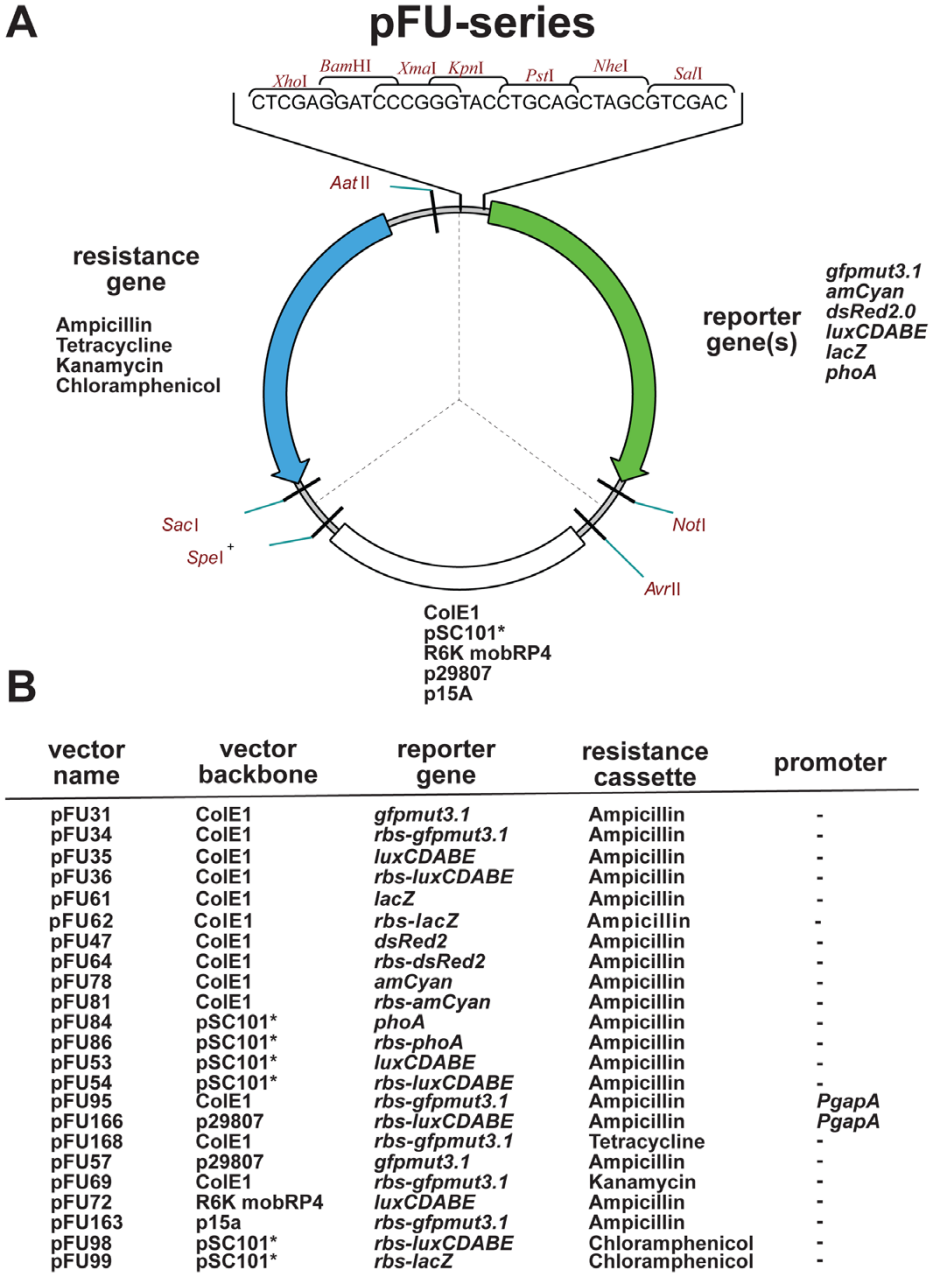
The pFU series of fusion vectors. (**A**) Composition of the fusion vectors of the pFU series. The vectors are made up of three interchangeable modules: (i) a resistance cassette with one of four different resistance genes encoded on a *Aat*II/*Sac*I fragment, (ii) a *Sac*I/*Avr*II fragment harboring one of five different origins of replication, and (iii) a *Xho*I/*Not*I fragment encoding the reporter gene with or without ribosome binding site downstream of a multiple cloning site containing seven unique restriction sites. +: *Spe*I site is not unique in plasmids carrying the pSC101* origin, (**B**) Representative collection of the pFU plasmids carrying the high-copy origin ColE1, the midi-copy origins p15A and p29807, the low-copy pSC101* origin, and the suicide vector R6K combined with a mobilizable origin (mob) for conjugation. They contain ampicillin, chloramphenicol, kanamycin, or tetracycline resistance genes including their promoter and ribosome binding sites which are oriented in the opposite direction of the reporter genes *luxCDABE*, *phoA*, *lacZ*, *gfp_mut3.1_*, *gfp_mut3.1_*-LVA, *amCyan* or *dsRed2* with or without their ribosome binding site (rbs).

The basic cloning vector of the pFU series (Figure **1**) was derived from vector pZE12-luc [21]. A fragment which contains the *gfp_mut3.1_* gene from pGFPmut3.1 (Clontech) was amplified by PCR with primers I916 and I917 including a new multiple cloning site (MCS) and inserted into the *Xho*I/*Xba*I sites of pZE12-luc to generate pFU31. The *dsRed2*, *amCyan*, *luxCDABE*, *lacZ*, and *phoA* reporter genes were PCR-amplified from different vectors (pDsRed2, pAmCyan, pUTmini-Tn5luxCDABEKm2, pHT124, pGP20) or genomic DNA derived from *E. coli* DH10beta(*phoA*) by using the primers listed in Figure **S2**.

All reporter and fluorescent genes were cloned into the *Sal*I/*Not*I site of pFU31 with or without an artificial ribosomal binding site (rbs), generating pFU34–38,47,64,78,81 (Figure **1**, Figures **S1**, **S2**). The *Sac*I site in the *lacZ* gene of pFU37 and pFU38 was exchanged by site-directed mutagenesis (GAGCTC→GAACTC) using the QuikChange site-directed mutagenesis kit (Stratagene) with primers II89 and II90 to construct plasmids pFU61 and pFU62. Replacement of the ColE1 origin of replication by the very low-copy origin of pSC101*, generating pFU51,53–54 or the midi-copy origin of p15A, generating pFU163, was performed by exchanging the *Sac*I/*Avr*II fragment against the *Sac*I/*Avr*II fragment of vectors pZS*24MCS and pZA31-luc, respectively. Replacement of the ColE1 origin of pFU31,34–35 against the origin of low-copy plasmid p29807, generating pFU57–59, was accomplished by exchanging *Spe*I/*Avr*II fragment against a *Spe*I/*Avr*II fragment amplified from vector pIV2mob with primers I953/I954. pFU68 resulted from pFU61 by an exchange of the *Sac*I/*Avr*II origin fragment of that of pZS*24MCS. The *phoA* gene with or without rbs was amplified with primers II143/II167 or II143/II168, and was directly cloned into the *Sal*I/*Not*I sites of pFU53, leading to pFU84 and pFU86. To employ the vector system for chromosomal integration, a PCR fragment amplified with primers II134/II135 of pGP704 containing the mobilization origin (mob) of plasmid RP4 in combination with the suicide origin R6K was inserted between the *Spe*I/*Avr*II sites of pFU35 for generation of pFU72. The kanamycin resistance gene encoded on an *Aat*II/*Sac*I fragment of pZS*24MCS was cloned into the *Aat*II/*Sac*I sites of plasmid pFU34, resulting in pFU69. pFU98 and pFU99 were constructed by insertion of the chloramphenicol resistance gene fragment of pZA31-luc into the *Xho*I/*Sac*I sites of pFU54 and pFU68, respectively. The tetracycline resistance gene was PCR amplified from pGP20 with primers I918/I970 and introduced in the *Sac*I/*Aat*II sites of pFU34 to generate pFU168. The promoter of the *E. coli gapA* gene was amplified with primers II147/II148 and inserted into the *Bam*HI and *Sal*I sites of pFU34, pFU64, pFU81 or pFU59 to construct plasmids that are constitutively expressing *gfp_mut3.1_* (pFU95), *dsRed2* (pFU96), *amCyan* (pFU97) and bacterial luciferase genes *luxCDABE* (pFU166). Vectors pFU221 and pFU224 were both constructed from vector pFU34. A *gfp_mut3.1_*-LVA fragment with or without rbs was amplified from vector pGFPmut3.1 with primers I952/II525 and I916/II525, respectively. The sequence of the LVA-tag was exchanged to the sequence of AAV- and ASV-tags resulting in vectors pFU222, pFU223, pFU225 and pFU226 using the QuikChange™ site-directed mutagenesis kit (Stratagene) with primers III30/III31 (AAV) or primers III32/III33 (ASV). To construct vector pKH59 the *rovA* promoter region was amplified with primers II258/II274 from genomic DNA of *Yersinia pseudotuberculosis* YPIII and integrated into the *Xho*I/*Pst*I sites of vector pFU31. Plasmid pKH83 was constructed by inserting a PCR derived *rovA* fragment amplified with primers 158/II525 from plasmid pYPL into the *Sal*I/*Not*I sites of pFU31. For pRS23 (*yadA*-*phoA*) construction, the *luxCDABE* operon encoded on a *Sal*I/*Not*I fragment of pTS31, was exchanged against the *Sal*I/*Not*I *phoA* reporter fragment of pFU86. Plasmids pTS31 (*yadA*-*luxCDABE*) and pTS42 (*yadA*-*lacZ*) were constructed by amplification of the *yadA* upstream region with primers 90/II308 from genomic DNA of *Y. pseudotuberculosis* YPIII and integrated into the *Bam*HI/*Sal*I sites of pFU98 and pFU99, respectively. Plasmid pTS36 was created of pTS31 by an exchange of the *Sac*I/*Spe*I fragment against that of pFU31 including the ColE1 origin. Plasmid pTS37 was derived from pTS31 by an exchange of the *Sac*I/*Not*I fragment of pFU57 harboring the p29807 origin. Plasmids pTS39 (*yadA*-*gfp_mut3.1_*) and pTS40 (*yadA*-*dsred2*) were engineered by an exchange of the *luxCDABE* operon encoded on a *Sal*I/*Not*I fragment of pTS37 against the *Sal*I/*Not*I reporter gene fragments of plasmids pFU31 and pFU64, respectively. Plasmid pTS43 was constructed by replacing the *Avr*II/*Spe*I fragment harboring the replication origin of pFU34 against the corresponding fragment of pZA31-luc carrying the replication origin of p15A. To construct plasmids pTS28 (*yopE-gfp_mut3.1_*) and pWO34 (*yopE*-*luxCDABE*) a PCR fragment was generated with primers II306/II307 from genomic DNA of *Y. pseudotuberculosis* YPIII and integrated into the *Bam*HI/*Sal*I sites of pFU58 and pFU98, respectively.

### Detection of the reporter proteins and analysis of the reporter gene expression under *in vitro* conditions

Bacteria harboring the fusion plasmids were diluted 1∶50 in fresh LB from overnight cultures and grown to late exponential phase at 25°C or 37°C. Beta-galactosidase and alkaline phosphatase activity were measured in cell free extracts as described previously [22]. The activities were calculated as follows: Beta-galactosidase activity OD_420_·6,75·OD_600_^−1^·Δt (min)^−1^·Vol (ml)^−1^ and alkaline phosphatase activity OD_420_·6,46·OD_600_^−1^·Δt (min)^−1^·Vol (ml)^−1^. Beta-galactosidase assays were performed in triplicate of cultures grown under indicated conditions. Reporter fusions emitting bioluminescence were measured in non-permeabilized cells with a Varioskan Flash (Thermo Scientific) using the SkanIt software (Thermo Scientific) for 1 s per time point and every 15 min for kinetic analyses. The data are given as relative light units (RLU/OD_600_) from three independent cultures performed in duplicate or are compared with the colony forming units of the cultures determined by plating. GFPmut3.1 and DsRed2 expressed in *Y. pseudotuberculosis* in culture media, in association with cells or within host tissues were also visualized with a fluorescence microscope (Axiovert II with Axiocam HR, Zeiss, Germany) using the AxioVision program (Zeiss, Germany).

To test transient expression events, *Y. pseudotuberculosis* YPIII harbouring a plasmid encoding an unstable variant of GFPmut3.1 (pFU31) was diluted 1∶50 in fresh LB from overnight cultures and grown at 25°C to exponential phase before the culture was shifted to 37°C. In addition,

### Monitoring of virulence gene expression in association with host cells

YPIII pTS28 harboring a *yopE*-*gfp_mut3.1_* fusion was grown in LB medium at 25°C overnight. About 10^3^ HEp-2 cells, seeded in a μ-slide (ibidi, Germany) were infected with 5×10^4^ bacteria. The bacteria were centrifuged onto the epithelial cells, incubated at 25°C to prevent internalization into host cells, and *yopE-gfp_mut3.1_* expression was followed over four hours by fluorescence microscopy as described above.

### *In vivo* monitoring of *yadA-gfp_mut3.1_* and *yadA-luxCDABE* expression during mouse infections

YPIII harboring a *yadA*-*gfp_mut3.1_* fusion (pTS39) and a *gapA*-*dsRed2* expression construct (pFU96) were grown in LB medium at 25°C overnight. 6–8 week old female Balb/c mice were orally infected with 1×10^9^ bacteria. After 3 days post infection, mice were sacrificed by CO_2_ asphyxiation. For cryosections, the small intestine and the mesenteric lymph nodes (MLNs) were embedded in Tissue-Tek OCT freezing medium (Sakura Finetek) and frozen on dry ice. Subsequently, 8–10 µm sections were prepared using a Zeiss cryostat Hyrax C50 and mounted on SuperFrost Plus slides (Thermo Scientific). Air-dried sections were fixed for 10 min in ice-cold acetone and washed twice with PBS. For visualization of the nuclei in the fixed tissue, sections were stained with 4′,6-diamidino-2-phenylindole (DAPI, Sigma) twice for 5 min, air-dried and mounted with 80% glycerol in PBS. Localization of the yersiniae in the infected tissues and expression of the *yadA-gfp_mut3.1_* fusion of these bacteria were visualized by a fluorescence microscope (Axiovert II with Axiocam HR, Zeiss, Germany) using the AxioVision program (Zeiss, Germany).

To monitor the course of a bacterial infection in mice and to detect virulence gene expression during the infection process *Y. pseudotuberculosis* strain YPIII pFU166 harboring a *gapA*-*luxCDABE* fusion or YPIII pTS31 expressing a *yadA*-*luxCDABE* fusion were grown in LB medium at 25°C overnight. About 1×10^9^ luminescent bacteria were used for oral infection of 6–8 weeks old female Balb/c mice. For *in vivo* imaging mice were anesthesized with isoflurane and the bacterial infection was followed daily using the IVIS Lumina system (Xenogen). At indicated time points after infection, some of the mice were sacrificed by CO_2_ asphyxiation. The intestinal tract, MLNs, liver and kidney were removed and also subjected to analysis by the IVIS Lumina system. To ensure maintenance of the plasmids during the course of the infection, the bacteria were isolated from the caecum and MLNs, and tested for the presence of the plasmid.

## Results and Discussion

### Composition and construction of the pFU vector family

In order to follow and quantify virulence gene expression under different growth conditions *in vitro*, in association with cells and during infection processes, we developed a series of compatible vectors based on the modular pZ expression plasmids [21]. The constructed promoter probe vectors contain interchangeable (a) fluorescent, bioluminescent and colorigenic reporter genes with multiple cloning sites, (b) resistance genes against four antibiotics, and (c) five different origins of replications allowing variation of the compatibility and copy number as well as chromosomal integration and long-term stability without antibiotic selection. The ColE1, the p15A and the modified pSC101 (pSC101*) origins of replication were derived from plasmids pZE, pZA and pZS* with intracellular copy numbers of 50–70, 20–30 and 3–4 [21]. The p29807 replication region was obtained from a plasmid p29807 of a *Yersinia enterocolitica* biogroup 1A strain with approximately 12–14 copies per cell [23], and the R6K-based origin for chromosomal integration of the reporter fusions was acquired from the mobilizable suicide vector pGP704 [24]. The origins are all encoded on *Avr*II-*Spe*I fragments that were used as backbones for the pFU series of promoter probe vectors (Figures **1**, **S1**). The pSC101* origin which contains an internal *Spe*I site can be exchanged as *Sac*I/*Avr*II fragment (Figure **1**). Furthermore, different resistance markers against the antibiotics ampicillin, kanamycin, chloramphenicol, and tetracycline encoded on *Sac*I/*Aat*II fragments from the pZ series or other vectors (Figure **S1**) were combined with the different vector backbones. The third module encoded on a *Xho*I/*Not*I fragment of the vector carries the reporter genes *gfp_mut3.1_* or unstable derivative *gfp_mut3.1_*-LVA/-AAV/-ASV, *dsRed2*, *amCyan*, *lacZ*, *phoA*, or the *luxCDABE* operon with their associated multiple cloning sites. The reporter genes were inserted with or without their ribosomal binding sites (rbs) to enable the construction of transcriptional and translational fusions. The GFPmut3.1 derivative with an excitation maximum of 501 nm and an emission maximum of 511 nm was shown to be about 35-fold more fluorescent than the GFP wild-type protein [6]. Exchange of this highly fluorescent variant against the less stable GFPmut3.1 variants allows detection of transiently expressed genes. In contrast to *gfp_mut3.1_*, the *amCyan* reporter gene encodes an autofluorescent 108 kDa protein (AmCyan) from *Anemonia majano* with an excitation maximum of 458 nm and an emission maximum of 489 nm and can be used for colony screening with an UV transilluminator [25], [26], [27]. The *dsRed2* reporter gene is a variant from the red fluorescent protein drFP583 of *Discosoma* sp. with excitation/emission maxima of 558/583 nm. It contains six amino acid exchanges that prevent protein aggregation and result in a more rapid appearance of the fluorescence [9]. . The DsRed2 protein can be used for multiplex applications, e.g. for simultaneous detection of two or more events in the same bacterium or a population *in vitro*, in association with host cells or within infected tissues. It can be used in a two- or three-color analysis with GFPmut3.1, DAPI or AmCyan, ideal for simultaneous monitoring of gene expression *in vitro* and *in vivo*. To quantify and compare gene expression with traditional colorigenic reporter genes, we also introduced the beta-galactosidase (*lacZ*) and the alkaline phosphatase (*phoA*) genes into the vector system. Many *lacZ* and *phoA* deletion mutants of *E. coli* and other bacteria are available for expression analysis [28]. Moreover, very low expression levels of equivalent genes in other bacteria allow use of these reporters in many wild-type strains, e.g. *yersiniae* [22]. For imaging light emission from the expression of luciferase reporter genes in living cells and organisms, we further cloned the *luxCDABE* operon of the terrestrial *Photorhabdus luminescence* into our vector system. This bacterial *lux* system is unique and particularly useful as a reporter system since all components (luciferase and substrates) for generating the bioluminescent signal are present within the bacterial cell and the self-generated response can directly be linked to photonic detectors facilitating *in vivo* monitoring.

The different reporter systems as well as the origin and antibiotic resistance modules of the FU plasmids can easily be exchanged using unique restriction sites and similar multiple cloning sites allow parallel constructions of different reporter fusions. Use of different origins allows expression analysis of genes with a very low or high promoter activity and permits simultaneous expression analysis of different genes. It is also often desirable to visualize bacteria during the course of an infection and study localization of different variants of pathogenic bacteria (e.g. wild-type and mutants) within the host tissues in co-infection experiments. Therefore, we developed an additional vector set in which the different autofluorescent and bioluminescent reporter genes are constitutively expressed. For this purpose we introduced the promoter of the constitutively expressed glyceraldehyde-3-phosphate-dehydrogenase (P*_gapA_*) upstream of the *gfp_mut3.1_*, *dsRed2*, *amCyan* and *luxCDABE* genes. The composition and properties of the available vectors are summarized in Figures **1** and **S1**. However, due to the modular make-up of the vector system it can be easily extended by the addition of other reporter genes, antibiotic resistance cassettes, additional origins of replication and promoter sequences to adapt and optimize it for gene expression studies with other microbes.

### Analysis of *Yersinia* virulence gene expression analysis *in vitro*

**Figure 2:**
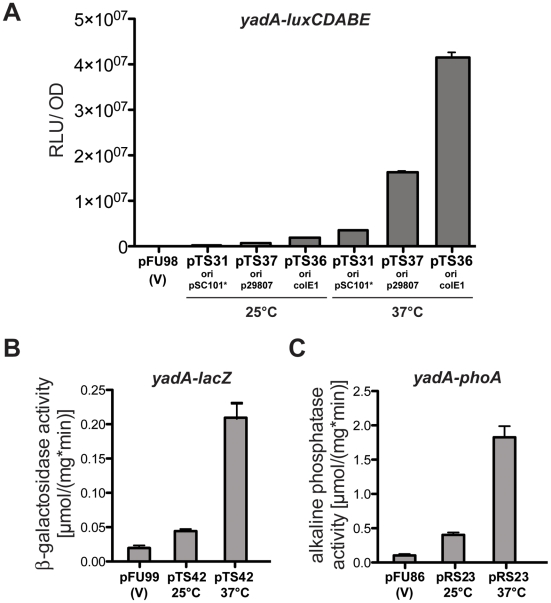
Comparative gene expression analysis using pFU plasmids. (**A**) *Y. pseudotuberculosis* strain YPIII harboring the empty vector pFU98 (V) or different *yadA-luxCDABE* reporter fusions encoded on a low copy pSC101* (pTS31), a midi-copy p29807 (pTS37) or a high-copy ColE1 based vector (pTS36) were diluted 1∶50 in fresh LB from overnight cultures and grown to late exponential phase at 25°C or 37°C. Bioluminescence was determined as described in Material and Methods and is given as relative light units (RLU) per optical density at 600 nm (OD). Expression of a *yadA-lacZ* (**B**) and a *yadA-phoA* (**C**) fusion encoded on pSC101*-based vectors in *Y. pseudotuberculosis* YPIII were determined from cultures grown under the identical conditions described above. YPIII harboring the empty vector plasmids were used as negative controls. Beta-galactosidase and alkaline phosphatase activity was determined with whole cell extracts. Data represent the mean of three independent experiments done in triplicate and are given in µmol mg^−1^ and min^−1^. The fluorescence of the cultures was measured as described in Material and Methods and is given as relative fluorescence units (RFU) per optical density at 600 nm (OD).

**Figure 3:**
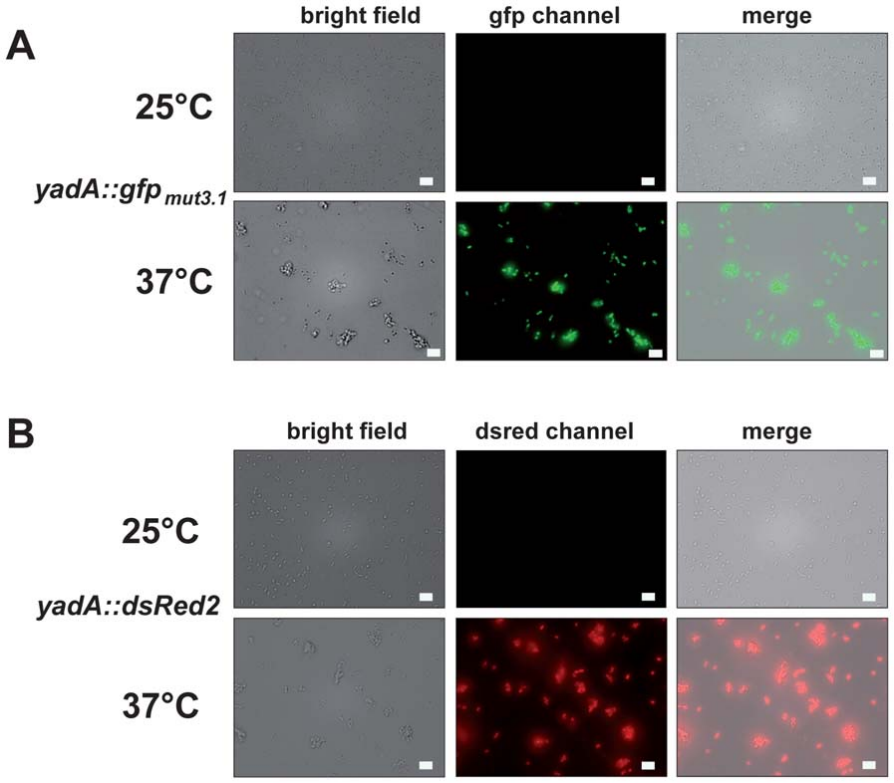
Comparative gene expression analysis using pFU plasmids. *Y. pseudotuberculosis* strains YPIII pTS39 and YPIII pTS40 were diluted 1∶50 in fresh LB from overnight cultures and grown to late exponential phase at 25°C or 37°C. Bacteria were detected by bright field microscopy and expression of the *yadA-gfp_mut3.1_* (**A**) and *yadA-dsRed2* (**B**) fusion was visualized by fluorescence microscopy. White bar indicates 5 µm.

It has previously been shown that expression of the *yadA* gene encoding the well-characterized *Yersinia* adhesin A is thermoregulated and is expressed only at 37°C by the temperature-regulated virulence gene activator LcrF [29], [30]. In order to test the different vectors, the *yadA* promoter region of *Y. pseudotuberculosis* strain YPIII was cloned into different pFU fusion vectors to generate transcriptional *yadA-luxCDABE* fusions on compatible low, medium and high copy vectors (pTS31, pTS37, pTS36). Furthermore, a pSC101*-based vector set was used to generate *yadA-lacZ*, *yadA-phoA*, *yadA-dsRed2* and *yadA*-*gfp_mut3.1_* fusions. The plasmids were transformed into *Y. pseudotuberculosis* strain YPIII and expression of the fusion was determined at 25°C (repressing conditions) and 37°C (inducing conditions) by bioluminescence, colorimetric and fluorescence detection assays (for details see Material and Methods). As shown in Figure **2A**, a 9–10fold difference in the *yadA* expression levels could be detected with the *luxCDABE* fusions (Figure **2A**), and a 4–5fold increase could be observed with the colorimetric reporters (Figure **2B–C**). In addition, both tested fluorescent reporters (*gfp_mut3.1_* and *dsRed2*) were highly expressed in the bacteria at 37°C, but no fluorescence was detectable at 25°C with the same photographic adjustments (Figure **3**). The fastest and most sensitive response with almost no background activity was observed with the *luxCDABE* reporter plasmids making these vectors very suitable for the analysis of weakly expressed genes. This is in agreement with a previous study demonstrating that the *luxCDABE* reporter is more reactive and achieves a more rapid response than the fluorescent proteins GFP and DsRed [31]. Furthermore, transcription levels of the *yadA-luxCDABE* reporter fusion were found to correlate with the copy number of the promoter probe vector (Figure **2A**). Ratio of temperature-dependent activation of *yadA* expression was very similar, but the expression level was strongly dependent on the copy-number of the fusion plasmids. A 2.5-fold higher expression was measured with the ColE1-based plasmid compared to the p29807 derivative, and a 3-fold higher activity was detectable with the p29807-based plasmid compared to the pSC101*-based vector, demonstrating that the vector system can be used for (simultaneous) expression analysis of genes with significantly different transcription levels. As bacterial luciferase activity depends on NADH and ATP, we also tested the influence of the metabolic activity of the bacterial cells on expression of the *luxCDABE* fusion system. To do so, we followed expression of a constitutive *gapA-luxCDABE* fusion expressed by *Y. pseudotuberculosis* YPIII pFU166 along the bacterial growth curve and compared luciferase activity with the optical density and the number of bacteria in the culture. As shown in Figure **S3**, luciferase activity increased during exponential phase reflecting the increase of the number of bacteria in the culture. However, bioluminescence declined continuously after the bacteria entered stationary phase, whereas an equivalent *gapA*-*gfp* fusion remained highly fluorescent (data not shown). This demonstrated that *luxCDABE* fusion systems are highly sensitive and very useful for the quantification of gene expression when the bacteria are metabolically active, whereas colorigenic and fluorescent reporter vector systems are more appropriate for the analysis of gene expression in resting/starved bacteria (e.g. during stationary phase).

**Figure 4:**
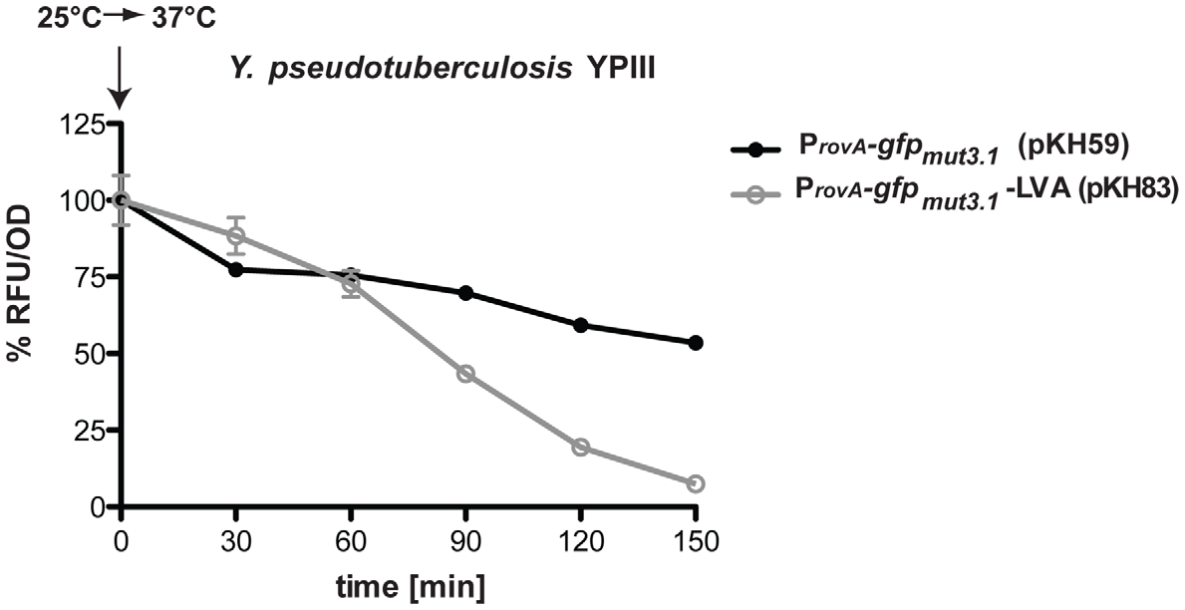
Comparison of *rovA-gfp_mut3.1_* and *rovA-gfp_mut3.1_*-LVA expression. *Y. pseudotuberculosis* strain YPIII pKH59 (*rovA-gfp_mut3.1_*) and YPIII pKH83 (*rovA-gfp_mut3.1_*-LVA) were grown overnight and fluorescence was determined as described in Material and Methods and is given as relative fluorescence units (RFU) per optical density at 600 nm (OD).

In order to allow the analysis of transient expression events, we also constructed a derivative of pFU31 encoding unstable variants of GFPmut3.1 tagged with a C-terminal extension, which is a target for tail-specific proteases [32]. Addition of an ASV-, AAV- and LVA -tag reduces the half-life of GFP_mut3.1_ from 24 hours to 110 min, 60 min and 40 min, respectively [33]. To demonstrate useful application of our vector system for the analysis of transient gene expression, we analyzed the activity of the *rovA* promoter of *Y. pseudotuberculosis* cloned upstream of the *gfp_mut3.1_* gene or the *gfp_mut3.1_*-LVA variant. It has been previously demonstrated that the *rovA* gene of *Y. pseudotuberculosis* encodes a MarR-type virulence regulator that acts as a protein thermometer [34]. A thermal upshift from 25°C to 37°C leads to a reversible conformational change of the regulator which reduces its DNA-binding function and renders it more susceptible for proteolysis. As a result cooperative binding to its own promoter region and autoactivation are significantly reduced at 37°C [34]. To test our vector system for transient gene expression *Y. pseudotuberculosis* YPIII carrying the *rovA-gfp_mut3.1_* or the *rovA*-*gfp_mut3.1_*-LVA fusion was grown at 25°C and shifted to 37°C for several hours. As shown in Figure **4**, *rovA*-*gfp_mut3.1_*-LVA expression was more rapidly repressed than the *rovA*-*gfp_mut3.1_* fusion after a thermal upshift from 25°C to 37°C. In addition to these experiments, we also monitored the luciferase activity of the constitutive *gapA-luxCDABE* fusion of *Y. pseudotuberculosis* YPIII pFU166 after transcription was stopped by the addition of rifampicin. As shown in Figure **S4**, luciferase activity declined rapidly after growth arrest, demonstrating that also the *luxCDABE* reporter is useful for the analysis of transient gene expression.

### Monitoring of *Yersinia* virulence gene expression upon host cell contact

**Figure 5:**
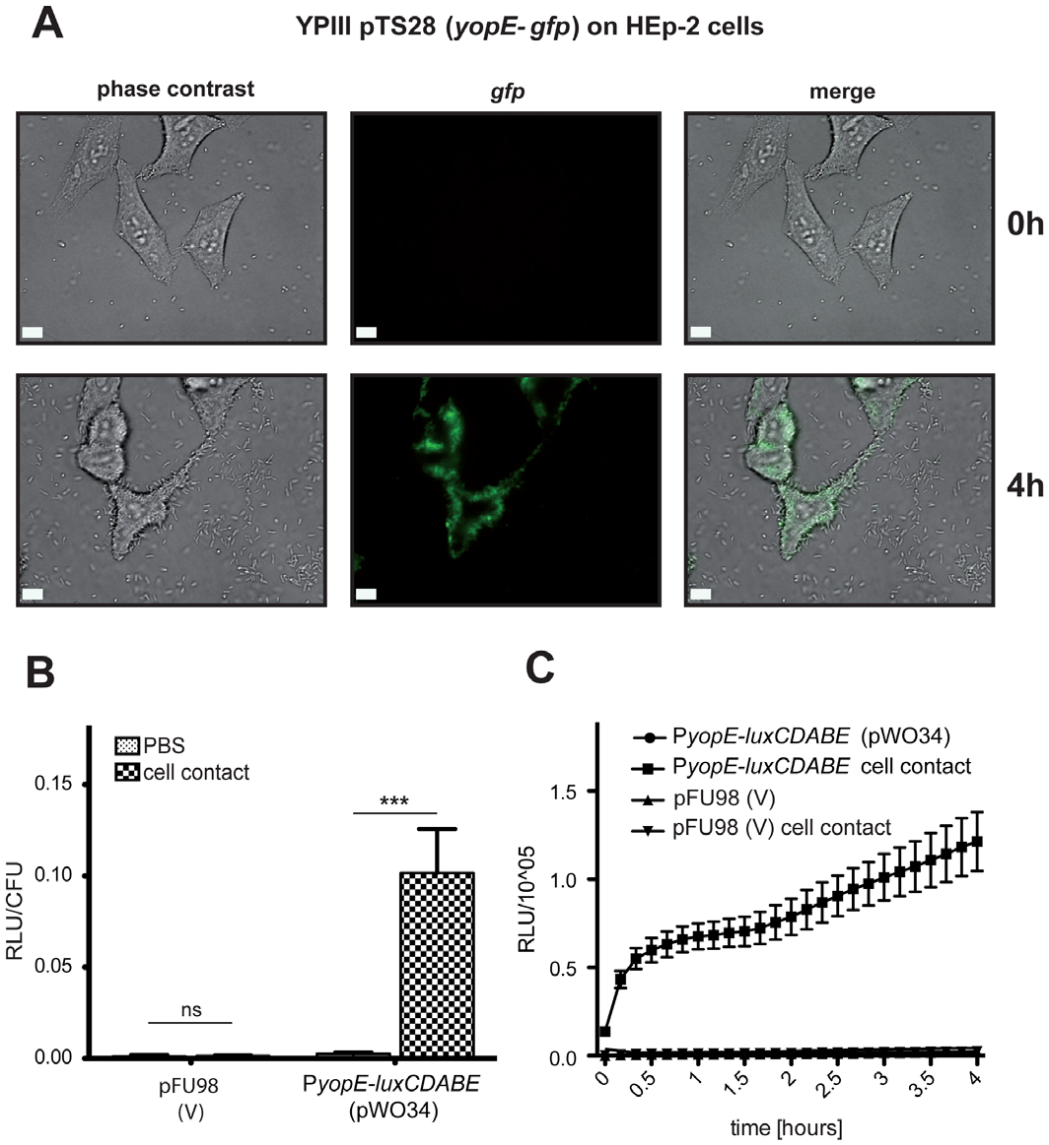
Cell contact dependent gene expression analysis using pFU plasmids. (**A**) About 5×10^4^ bacteria of *Y. pseudotuberculosis* strain YPIII pTS28 (*yopE-gfp_mut3.1_*) were used to infect 10^3^ HEp-2 cells. Bacteria were centrifuged onto the cell monolayer. Expression of the *yopE-gfp_mut3.1_* fusion was monitored by phase contrast and fluorescence microscopy directly after centrifugation (0 h) and after four hours (4 h). (**B**,**C**) About 10^6^ bacteria of *Y. pseudotuberculosis* strain YPIII pFU98 (empty vector) and YPIII pWO34 (*yopE-luxCDABE*) resuspended in PBS were incubated in the absence or in the presence of 10^4^ HEp-2 cells for 4 hours as described above. Bioluminescence emitted by cultures was monitored after 4 hours of infection (**B**), or was determined every 20 min after infection to monitor the kinetic of host cell contact-dependent *yopE-luxCDABE* induction (**C**). Bioluminescence is given as relative luminescence units (RLU) per colony forming units (CFU) and represents the mean of three independent experiments done in triplicate. White bar indicates 5 µm.

Autofluorescent and bioluminescent proteins are excellent reporters for monitoring gene expression in single bacteria in association with host cells, and they were successfully used to study bacterial gene expression within or onto host cells [35], [36]. For example, expression of the pathogenicity factor YopE of *Yersinia* was shown to be induced upon host cell contact [35]. In order to test our reporter plasmids we challenged cells with YPIII pTS28 and determined *yopE-gfp_mut3.1_* expression for four hours after infection. We found that yersiniae bound to HEp-2 cells were significantly more fluorescent than bacteria without host cell contact (Figure **5A**). A quantitative analysis of HEp-2 cells infected with YPIII harboring the *yopE-luxCDABE* fusion gave similar results and demonstrated a very strong (28-fold) increase of luciferase activity in the presence of host cells four hours post infection (Figure **5B**). A kinetic analysis of host cell contact-dependent activation of the *yopE-luxCDABE* fusion revealed a very strong increase of *yopE* expression during the first 30 min after infection which is followed by a smaller but steady increase of *yopE* expression (Figure **5C**). This clearly demonstrates that the different vector systems harboring fluorescent and bioluminescent reporter genes are suitable to follow and quantify gene expression of bacteria in association with cells.

### Monitoring of *Yersinia* virulence gene expression during infection

Fluorescent and luminescent reporter proteins are also very useful for monitoring virulence gene expression in infected tissues during different stages of the infection. However, stable maintenance of the autofluorescence and bioluminescence vectors is necessary for *in vivo* studies and this usually requires concomitant antibiotic selection that can interfere with the infection process, in particular during intestinal colonization. Maintenance of the plasmids harboring the backbone of pSC101*, p29807, p15A, and ColE1 was tested in *E. coli* MC4100, *Y. pseudotuberculosis* YPIII and *S. typhimurium* SL1344 grown for 21 days by replica plating of a culture aliquot on media containing no and the appropriate antibiotic (Figure **S4**). All three enteric strains retained the plasmids in >95% of the bacteria recovered from 6 days old cultures grown in complex media without antibiotics. However, after longer times of cultivation and nutrient deprivation, an increasing loss of the plasmids has been observed. This is particularly evident with the plasmids constitutively expressing the ATP-consuming luciferase (Figure **S5**). Accordingly, the *luxCDABE* expression vector can be used for infection experiments lasting up to one week, but it is preferable to integrate the fusion construct into the bacterial genome for long-term *in vivo* analysis, using the suicide R6K-based vector backbone.

**Figure 6:**
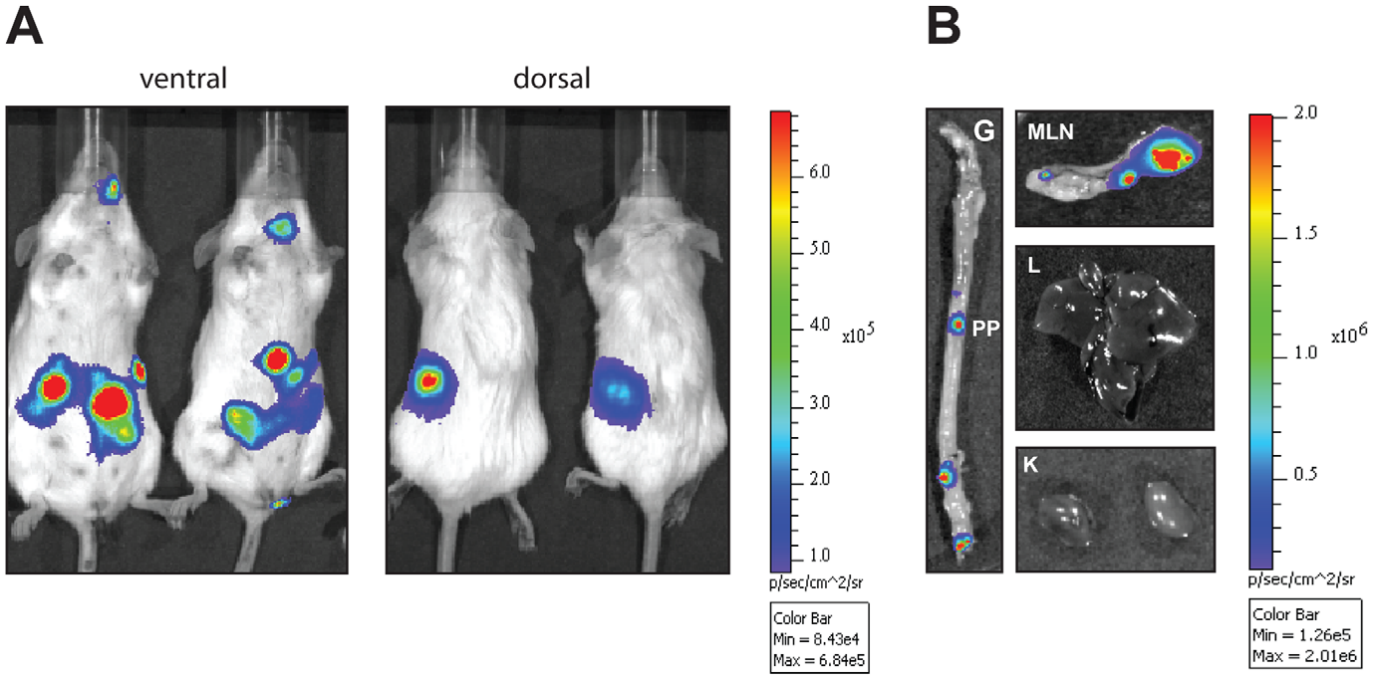
*In vivo* expression analysis of a *yadA-luxCDABE* fusion. (**A**) 1×10^9^ bacteria of *Y. pseudotuberculosis* strain YPIII pTS31 (*yadA-luxCDABE*) were used to orally infect Balb/c mice. Three days post infection, mice were anesthesized and bioluminescence was determined with an IVIS camera on the ventral or dorsal side. (**B**) Subsequently, mice were sacrifized and the gastrointestinal tract (G), the mesenterial lymph nodes (MLN) and the organs such as kidneys (K) and liver (L) were prepared and bioluminescence was determined with the IVIS system.

*Y. pseudotuberculosis* is known to replicate in lymphatic tissues (e.g. Peyer's patches (PPs), MLNs) and organs such as spleen, and the YadA adhesin was found to be crucial for the colonization of these tissues and organs [29]. In order to test our luminescent reporter plasmids expression of the *yadA* virulence gene was analyzed during infection of mice. To do so, we orally infected Balb/c mice with 1×10^9^ bacteria of the *Y. pseudotuberculosis* strain YPIII pTS31 harboring the *yadA-luxCDABE* fusion. Three days after infection mice were anesthetized and their bioluminescence was measured using an IVIS camera. As shown in Figure **6A** *yadA-luxCDABE* expression was detected in the intestinal tract and spleen demonstrating *yadA* expression in the bacteria during murine infections. Interestingly, the nasal cavity and the cervical lymph nodes of the mice were also luminescent after 3 days of infection, indicating colonization and *yadA* expression of the nasal-associated lymphoid tissue (NALT) by *Y. pseudotuberculosis*. In order to analyze colonization of the mice in more detail, mice were sacrificed at day three post infection and the small intestine, the MLNs, kidney and liver were removed and analyzed using the IVIS system. We found that multiple PPs were luminescent and luminescent bacteria were also observed within the MLNs (Figure **6B**). In contrast, no bioluminescence was detectable in mice infected with empty *luxCDABE* fusion plasmids (data not shown).

### Monitoring of a bacterial infection using the bioluminescent expression vector pFU166

**Figure 7:**
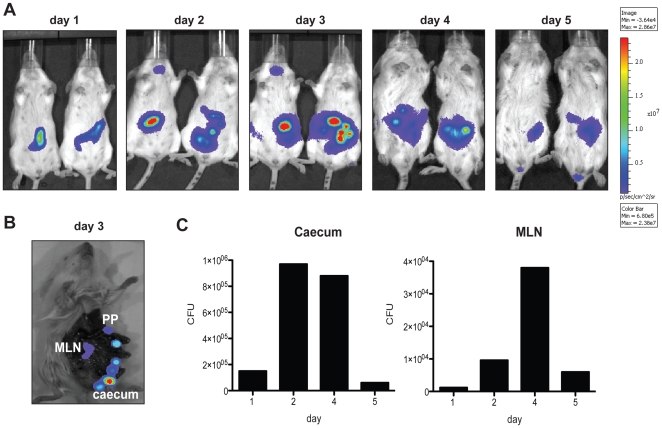
Monitoring of a *Y. pseudotuberculosis* YPIII infection expressing a constitutive *gapA*-*luxCDABE* fusion. 1×10^9^ bacteria of *Y. pseudotuberculosis* strain YPIII pFU166 (*gapA*-*luxCDABE*) were used to orally infect Balb/c mice. (**A**) 1–5 days post infection, mice were anesthesized and bioluminescence was determined with an IVIS camera on the ventral side. (**B**) On day 3, one mouse was sacrificed and bioluminescence of the gastrointestinal tract, including Peyer's patches (PP), caecum, and associated mesenterial lymph nodes (MLN) were analyzed with the IVIS system. (**C**) At the indicated time points 3 mice were killed, the caecum and the MLNs were prepared, homogenized and the number of bacteria in the tissues (CFU) per g organ were determined.

Vector pFU166 in which the bioluminescent reporter genes *luxCDABE* were constitutively expressed under the control of P*_gapA_* was used to follow the course of a *Yersinia* infection in Balb/c mice. A set of mice was intragastrically inoculated with 1×10^9^ bacteria of *Y. pseudotuberculosis* strain YPIII pFU166 (*gapA*-*luxCDABE*), and the bioluminescent signal was monitored in living animals for 5 days after infection. One day post-feeding a strong bioluminescent signal was observed in the intestinal tract of the infected mice. The signal increased to a plateau at day 3–4 and decreased again at day 5 (Figure **7A**). Preparation of the intestinal tract and gut-associated tissues further showed that the highest bioluminescent signal occured in the PPs, the MLNs and the caecum (Figure **7B**). In parallel, we determined the colony forming units of bacteria isolated from the intestinal tract (caecum) and the MLNs (Figure **7C**). Consistently to the bioluminescent intensity pictures, the highest number of bacteria was recovered from the caecum 2–4 days and from the MLNs 4 days post infection. These results indicated that colonization of the intestine and associated lymphatic tissues can be detected directly by simple recording of the bioluminescent signal in living animals harboring the *luxCDABE* expression plasmid. This constitutes an advantage compared to other inducible systems such as the P*_lacZ_*- or P*_xylE_*-dependent fusion constructs or the P*_BAD_*-*luxCDABE* expression system in which 150 mg L-arabinose needs to be applied intraperitoneally to induce luciferase expression 2–5 hours before monitoring of the luciferase activity during infection [37], [38].

**Figure 8:**
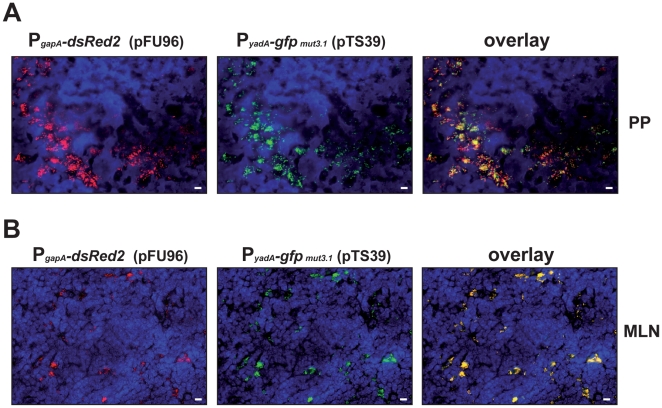
Detection of *Yersinia* in infected tissue and simultaneous analysis of *yadA-gfp_mut3.1_* expression. 1×10^9^ bacteria of *Y. pseudotuberculosis* strain YPIII pFU96/pTS39 (*gapA*-*dsRed2*; *yadA-gfp_mut3.1_*) were used to orally infect Balb/c mice. 3 days post infection, mice were sacrificed, PPs and MLNs were isolated. Histological slides were prepared and analyzed by fluorescence microscopy to detect the bacteria in the tissues by expression of the reporter protein DsRed2. In parallel *yadA* expression in the bacteria was monitored by GFPmut3.1 mediated fluorescence. White bar indicates 10 µm.

In order to identify bacteria in host tissues and study virulence gene expression in parallel, YPIII harboring pFU96 (*gapA*-*dsRed2*) and a compatible *yadA-gfp_mut3.1_* fusion plasmid (pTS39) was used to infect Balb/c mice. Cryosections were prepared of PPs and MLNs 3 days post infection. The bacteria in the tissues were visualized by monitoring DsRed2, and then tested for *yadA-gfp_mut3.1_* expression with a fluorescent microscope. As shown in Figure **8**, red-fluorescent bacteria could easily be detected within the tissues. By switching the fluorescence filter we could demonstrate that bacteria identified in these tissues also express the *yadA-gfp_mut3.1_* fusion construct (Figure **8**). Interestingly, *yadA* expression was highly induced in many, but not in all bacteria detectable in the PPs, indicating heterogeneous *yadA* expression, whereas all *yersiniae* in the microcolonies found in the MLNs expressed *yadA*.

In conclusion, our experiments demonstrate that the autofluorescent and luminescent reporter and expression vectors developed here are powerful tools to analyze and quantify colonization and gene expression of *Enterobacteriaceae* in infected cells and tissues of mice. By combining two different compatible autofluorescent reporters, bacteria can be detected within infected cells/tissues and expression of a particular bacterial gene or operon can be analyzed. Furthermore, it can be used for monitoring intestinal and organ colonization in whole living animals. The analysis of two bacterial populations with different reporter constructs allows simultaneous characterization of the expression of different genes and offers a simple method to address competition between wild-type and mutant strains. Construction of certain *gapA*-*luxCDABE* reporter plasmids allows *in vivo* monitoring of intestinal colonization and dissemination to deeper tissues/organs without antibiotic selection pressure and killing of the animals. In summary, this reporter and expression system has numerous potential applications for monitoring the initiation and progress of a bacterial infection and analyzing virulence gene expression during the infection process.

## Supporting Information

Figure S1 **Strains and plasmids.**
(DOC)

Figure S2 **Cloning and sequencing primers.**
(DOC)

Figure S3 **Analysis of** ***gapA-luxCDABE*** **expression along the bacterial growth curve.** *Y. pseudotuberculosis* YPIII pFU166 was diluted 1∶100 grown in LB medium at 37°C to late stationary phase. Every hour, an aliquot of the culture was removed, luminescence (relative light units - RLU) and optical density (OD) at 600 nm was determined, and dilutions of the aliquot were plated onto LB to determine the colony forming units (CFU) in the culture at the indicated time point. The RLU versus OD is given in **A**, and RLU versus CFU is illustrated in **B**.
(TIF)

Figure S4 **Analysis of the kinetic of** ***gapA-luxCDABE*** **expression.** *Y. pseudotuberculosis* YPIII pFU166 was diluted 1∶100 in LB medium and grown at 37°C to exponential phase (OD_600_ = 0.6). Subsequently, rifampicin (0.5 µg/ml) was added to block transcription. Every 15 min, an aliquot of the culture was removed and luminescence (relative light units - RLU) and optical density (OD_600_) was determined.
(TIF)

Figure S5 **Stability of the pFU plasmids in** ***Enterobacteriaceae*** **without antibiotic selection.** *E. coli* MC4100, *Y. pseudotuberculosis* YPIII and *S. typhimurium* SL3144 harboring plasmids pFU31, pFU51, pFU57, pFU166 or pTS43 were grown for 21 days in LB medium without antibiotics. Every day, 50% of the culture was inoculated with the identical volume of fresh LB medium. At indicated time points, an aliquot of the culture was removed and plated onto LB with and without antibiotics to test for the presence of the fusion plasmids.
(TIF)
