# Supplementary material for: Monitoring of Gene Expression in Bacteria during Infections Using an Adaptable Set of Bioluminescent, Fluorescent and Colorigenic Fusion Vectors
Source: PLoS One. 2011 Jun 3;6(6):e20425. doi: 10.1371/journal.pone.0020425 (PMC3108616; doi:10.1371/journal.pone.0020425)
Supplement: Figure S1 — Strains and plasmids. (DOC) [file pone.0020425.s001.doc]

**Figure S1:**

| **Strain or plasmid** | **Description** | **Source/reference** |
| --- | --- | --- |
| **Strains** |  |  |
| *E. coli* DH10β | Cloning and expression strain | Invitrogen |
| *E. coli* CC118 λpir | F^-^ ∆(*ara-leu*)7697 ∆(*lacZ*)74 ∆(*phoA*)20 *araD139 galE galK thi rpsE rpoB arfE*^am^ *recA1* | [38] |
| *E. coli* MC4100 | F^-^ *[araD139]_B/r_*Δ(*argF-lac*)*U169* λ^-^ e14^-^ *flhD5301* Δ(*fruK-yeiR*)725(*fruA25*) *relA1* *rpsL150*(*strR*) *rbsR22* Δ(*fimB-fimE*)632(::IS*1*) *deoC1* | [39] |
| S17-1 λpir | Tp^r^ Sm^r^ *recA*, *thi*, *pro*, *hsdR*^-^*M*^+^ RP4:2-Tc:Mu:Km Tn7, λpir | [40] |
|  |  |  |
| *Yersinia pseudotuberculosis*  YPIII pIB1 |  | [41] |
|  |  |  |
| *S. typhimurium* SL1344 |  | [42] |
|  |  |  |
| **Plasmids** |  |  |
| pAmCyan | Ap^r^; *amCyan* expression vector, ColE1 | Clontech |
| pDsRed2 | Ap^r^; *dsRed2* expression vector | Clontech |
| pFU31 | Ap^r^; *gfp_mut3.1_*; ColE1 | This work |
| pFU34 | Ap^r^; *rbs*-*gfp _mut3.1_*; ColE1 | This work |
| pFU35 | Ap^r^; *luxCDABE*; ColE1 | This work |
| pFU36 | Ap^r^; *rbs*-*luxCDABE*; ColE1 | This work |
| pFU37 | Ap^r^; *lacZ* ColE1 | This work |
| pFU38 | Ap^r^; *rbs*-*lacZ*; ColE1 | This work |
| pFU47 | Ap^r^; *dsRed2*, ColE1 | This work |
| pFU51 | Ap^r^; *gfp_mut3.1_*; pSC101* | This work |
| pFU53 | Ap^r^; *luxCDABE*; pSC101* | This work |
| pFU54 | Ap^r^; *rbs*-*luxCDABE*; pSC101* | This work |
| pFU57 | Ap^r^; *gfp_mut3.1_*; p29807 | This work |
| pFU58 | Ap^r^; *rbs*-*gfp_mut3.1_*; p29807 | This work |
| pFU59 | Ap^r^; *luxCDABE*; p29807 | This work |
| pFU61 | Ap^r^; *lacZ* ColE1 (*Sac*I-site removed) | This work |
| pFU62 | Ap^r^; *rbs*-*lacZ*; ColE1 (*Sac*I-site removed) | This work |
| pFU64 | Ap^r^; *rbs*-*dsRed2*; ColE1 | This work |
| pFU68 | Ap^r^; *rbs*-*lacZ*; pSC101* | This work |
| pFU69 | Kn^r^; *rbs*- *gfp_mut3.1_*; ColE1 | This work |
| pFU72 | Ap^r^; *luxCDABE*; R6K mobRP4 | This work |
| pFU78 | Ap^r^; *amCyan*; ColE1 | This work |
| pFU81 | Ap^r^; *rbs*-*amCyan*; ColE1 | This work |
| pFU84 | Ap^r^; *phoA*; pSC101* | This work |
| pFU86 | Ap^r^; *rbs*-*phoA*; pSC101* | This work |
| pFU95 | Ap^r^; *gapA*-*rbs-gfp_mut3.1_*; ColE1 | This work |
| pFU96 | Ap^r^; *gapA-rbs-dsred2*; ColE1 | This work |
| pFU97 | Ap^r^; *gapA-rbs-amCyan*; ColE1 | This work |
| pFU98 | Cm^r^; *rbs*-*luxCDABE*; pSC101* | This work |
| pFU99 | Cm^r^; *rbs*-*lacZ*; pSC101* | This work |
| pFU163 | Ap^r^; *rbs*-*gfp_mut3.1_*; p15A | This work |
| pFU166 | Ap^r^; *gapA-rbs-luxCDABE*; p29807 | This work |
| pFU168 | Tet^r^; *rbs*-*gfp_mut3.1_*; ColE1 | This work |
| pFU221 | Ap^r^, *rbs*-*gfp_mut3.1_-*LVA; ColE1 | This work |
| pFU222 | Ap^r^, *rbs*-*gfp_mut3.1_-*AAV; ColE1 | This work |
| pFU223 | Ap^r^, *rbs*-*gfp_mut3.1_-*ASV; ColE1 | This work |
| pFU224 | Ap^r^, *gfp_mut3.1_-*LVA; ColE1 | This work |
| pFU225 | Ap^r^, *gfp_mut3.1_-*AAV; ColE1 | This work |
| pFU226 | Ap^r^; *gfp_mut3.1_-*ASV; ColE1 | This work |
| pGFPmut3.1 | *gfp_mut3.1_* expression vector | Clontech |
| pGP20 | Tet^r^, *lacZ*; pSC101 | Petra Gerlach |
| pGP704 | Ap^r^; cloning vector; R6K | [24] |
| pHT124 | Ap^r^; probe vector, *lacZ* with rbs | H. Tran Winkler |
| pIV2mob | Km^r^; expression vector; ori29807 | [23] |
| pKH59 | Ap^r^; *rovA*-*gfp_mut3.1_*; ColE1 | This work |
| pKH83 | Ap^r^; *gfp_mut3.1_*-LVA; ColE1 | This work |
| pRS23 | Cm^r^; *yadA*-*phoA*; pSC101* | This work |
| pTS28 | Ap^r^; *yopE*-*gfp_mut3.1_*; p29807 | This work |
| pTS31 | Cm^r^; *yadA*-*luxCDABE*; pSC101* | This work |
| pTS36 | Cm^r^; *yadA*-*luxCDABE*; ColE1 | This work |
| pTS37 | Cm^r^; *yadA*-*luxCDABE*; p29807 | This work |
| pTS39 | Cm^r^; *yadA*-*gfp_mut3.1_*; p29807 | This work |
| pTS40 | Cm^r^; *yadA*-*dsred2*; p29807 | This work |
| pTS42 | Cm^r^; *yadA*-*lacZ*; pSC101* | This work |
| pTS43 | Ap^r^; *rbs*-*gfp _mut3.1_*; p15A | This work |
| pUTmini-Tn5luxCDABEKm2 | Km^r^; Tn5-*luxCDABE*; R6K | [43] |
| pWO34 | Cm^r^; *yopE*-*luxCDABE*; pSC101* | This work |
| pYPL | Km^r^; *rovA*-*gfp _mut3.1_*-LVA; pPROBE | [44] |
| pZE12-luc | Ap^r^; PLlacO-1::*luc*; ColE1; | [21] |
| pZA31-luc | Cm^r^; PLtetO-1::*luc;* p15A, | [21] |
| pZS*24MCS | Km^r^; Plac/ara-1; pSC101* | [21] |

*rbs*: ribosome binding site
