# Supplementary material for: Monitoring of Gene Expression in Bacteria during Infections Using an Adaptable Set of Bioluminescent, Fluorescent and Colorigenic Fusion Vectors
Source: PLoS One. 2011 Jun 3;6(6):e20425. doi: 10.1371/journal.pone.0020425 (PMC3108616; doi:10.1371/journal.pone.0020425)
Supplement: Figure S2 — Cloning and sequencing primers. (DOC) [file pone.0020425.s002.doc]

**Table S2:** Cloning and sequencing primers

| **Primer** | **Sequence** | **Gene/ promoter** | **Plasmid** | **Template** |
| --- | --- | --- | --- | --- |
| 90 | CGC GGC GGA TCC CCT TAA CGT ACG TTT TCG TCC C | P*_yadA_* | pTS31 | Genomic DNA YPIII |
| 158 | GCC GCG GTC GAC TGC CGC CTT CCT GCA ACT CG | P*_rovA_* | pKH59 | Genomic DNA YPIII |
| I916 | GCA CGC TCG AGG ATC CCG GGT ACC TGC AGC TAG CGT CGA CAT GCG TAA AGG AGA AG | *gfpmut3.1*+MCS *gfpmut3.1-*LVA | pFU31, 221 | pGFPmut3.1 |
| I917 | GCA CGT CTA GAG CGG CCG CTT ATT TGT ATA GTT CAT CCA TGC C | *gfpmut3.1* | pFU31,34 | pGFPmut3.1 |
| I918 | GCA CTG AGC TCA ATC CGT TAG CGA GGT GCC | Tet^R^ | pFU32 | pGP20 |
| I920 | GCA CGG TCG ACA TGA CTA AAA AAA TTT CAT TCA TTA TTA AC | *luxCDABE* | pFU35 | pUTmini-Tn5luxCDABEKm2 |
| I921 | GCA CTG CGG CCG CTC AAC TAT CAA ACG CTT CGG TTA AG | *luxCDABE* | pFU35,36 | pUTmini-Tn5luxCDABEKm2 |
| I922 | GCA CTG TCG ACG TCG TTT TAC AAC GTC GTG ACT G | *lacZ* | pFU37 | pGP20 |
| I923 | GCA CGG CGG CCG CTT ATT TTT GAC ACC AGA CCA ACT G | *lacZ* | pFU37,38 | pGP20, pHT124 |
| I924 | GCA CGG TCG ACA GGA GGA ATT AAA AAT GAA AGG G | *lacZ* | pFU38 | pHT124 |
| I951 | GCA CGG TCG ACA GGA GGA TAC GTA TGA CTA AAA AAA TTT C | *luxCDABE* | pFU36 | pUTmini-Tn5luxCDABEKm2 |
| I952 | GCA CGG TCG ACG AGG AGA AAT TAA GCA TGC G | *gfpmut3.1* | pFU34 | pGFPmut3.1 |
| I953 | GCA CGC CTA GGC GGA GAT ATT CCG CTT CCT CG | ori p29807 | pFU57 | pIV2mob |
| I954 | GCA CGA CTA GTA AGA TGA ACC TCT TGA GAT CC | ori p29807 | pFU57 | pIV2mob |
| I970 | GCA CGG ACG TCT CAT GTT TGA CAG CTT ATC ATC | Tet^R^ | pFU32 | pGP20 |
| I984 | TAA GAA ACC ATT ATT ATC ATG AC | sequencing |  |  |
| I985 | GTG AAG ACG AAA GGG CCT C | sequencing |  |  |
| I986 | GGC ATC AAA TAA AAC GAA AG | sequencing |  |  |
| I987 | TCT AGG GCG GCG GAT TTG | sequencing |  |  |
| I988 | TGG ATT CTC ACC AAT AAA AAA C | sequencing |  |  |
| I989 | GCT TGG ACT CCT GTT GAT AG | sequencing |  |  |
| II26 | GCA CGG TCG ACA TGG CCT CCT CCG AGA AC | *dsRed2* | pFU47 | pDsRed2 |
| II27 | GCA CTG CGG CCG CCT ACA GGA ACA GGT GGT GG | *dsRed2* | pFU47,64 | pDsRed2 |
| II70 | GCA CGG TCG ACA GAG GAA TTA ACC ATG GCC TCC TCC GAG | *dsRed2* | pFU64 | pDsRed2 |
| II89 | CAT AGC-GAT AAC GAA CTC CTG CAC TGG ATG | mutagenesis | pFU61,62 | pFU37, pFU38 |
| II90 | CAT CCA GTG CAG GAG TTC GTT ATC GCT ATG | mutagenesis | pFU61,62 | pFU37, pFU38 |
| II134 | GCA CGC CTA GGC ACA AAT TGT TAT CCG CTC | ori mobRP4/R6K | pFU100 | pGP704 |
| II135 | GCA CGA CTA GTG GAT CCT TTT TGT CCG G | Ori mobRP4/R6K | pFU100 | pGP704 |
| II138 | GCA CGG TCG ACA GGA GGA ATT AAC CAT GGC TCT TTC AAA CA | *amCyan* | pFU81 | pAmCyan |
| II139 | GCA CGG TCG ACA TGG CTC TTT CAA ACA AGT | *amCyan* | pFU78 | pAmCyan |
| II140 | GAC ATG CGG CCG CTT CAG AAA G | *amCyan* | pFU78,81 | pAmCyan |
| II143 | GCA CTG CGG CCG CTT ATT TCA GCC CCA GAG C | *phoA* | pFU84,86 | Genomic DNA *E.coli* DH10ß |
| II147 | GCA CGG GAT CCG CCA TTT GCT CAC ATC TC | P*_gapA_* | pFU95-97 | Genomic DNA *E.coli* DH10ß |
| II148 | GCA CGG TCG ACA TAT TCC ACC AGC TAT TTG TTA G | P*_gapA_* | pFU95-97 | Genomic DNA *E.coli* DH10ß |
| II167 | GCA CGG TCG ACA TGA AAC AAA GCA CTA TTG CAC | *phoA* | pFU86 | Genomic DNA *E.coli* DH10ß |
| II168 | GCA CGG TCG ACA GGA GGA ATT AAC CAT GAA ACA AAG CAC TAT TGC AC | *phoA* | pFU84 | Genomic DNA *E.coli* DH10ß |
| II258 | GCG CGC CTC GAG CCA ATC GCG CTC AGA CTG GC | P*_rovA_* | pKH59 | Genomic DNA YPIII |
| II274 | GCG CGC CTG CAG TTA TTA ATC AGA TCC TAA TGT CGA TTC CAA | *rovA*-*gfp_mut3.1_-*LVA | pKH83 | pYPL |
| II306 | GCA CTG GAT CCT AGT ATC TGG AAT AGA CAA CGA AAG | *P_yopE_* | pWO34 | Genomic DNA YPIII |
| II307 | GCA CTG TCG ACT TAT ATT TTC ATG ACT ATT TAT TAC CTT GG | *P_yopE_* | pWO34 | Genomic DNA YPIII |
| II308 | CGC GGC GTC GAC TTA CGC AGA TAT TAA TGC CGC AGA GAC | P*_yadA_* | pTS31 | Genomic DNA YPIII |
| II525 | GCG CGC GCG GCC GC TTA AGC TAC TAA AGC GTA GTT TTC GTC GTT TGC TGC TTT GTA TAG TTC ATC CAT GCC ATG | *rovA*-*gfp_mut3.1_-*LVA | pKH83, pFU221, 224 | pYPL, pGFPmut3.1 |
| III30 | AAC GAC GAA AAC TAC GCT GCT GCT GTA TAA GCG GCC GCT CTA GAG | mutagenesis | pFU222, 225 | pFU221, 224 |
| III31 | CTC TAG AGC GGC CGC TTA TAC AGC AGC AGC GTA GTT TTC GTC GTT | mutagenesis | pFU222, 25 | pFU221, 224 |
| III32 | AAC GAC GAA AAC TAC GCT GCT AGC GTA *TAA* GCG GCC GCT CTA GAG | mutagenesis | pFU223, 226 | pFU221, 224 |
| III33 | CTC TAG AGC GGC CGC TTA TAC GCT AGC AGC GTA GTT TTC GTC GTT | mutagenesis | pFU223, 226 | pFU221, 224 |
