# Supplementary figures and images for: Monitoring of Gene Expression in Bacteria during Infections Using an Adaptable Set of Bioluminescent, Fluorescent and Colorigenic Fusion Vectors
Source: PLoS One. 2011 Jun 3;6(6):e20425. doi: 10.1371/journal.pone.0020425 (PMC3108616; doi:10.1371/journal.pone.0020425)

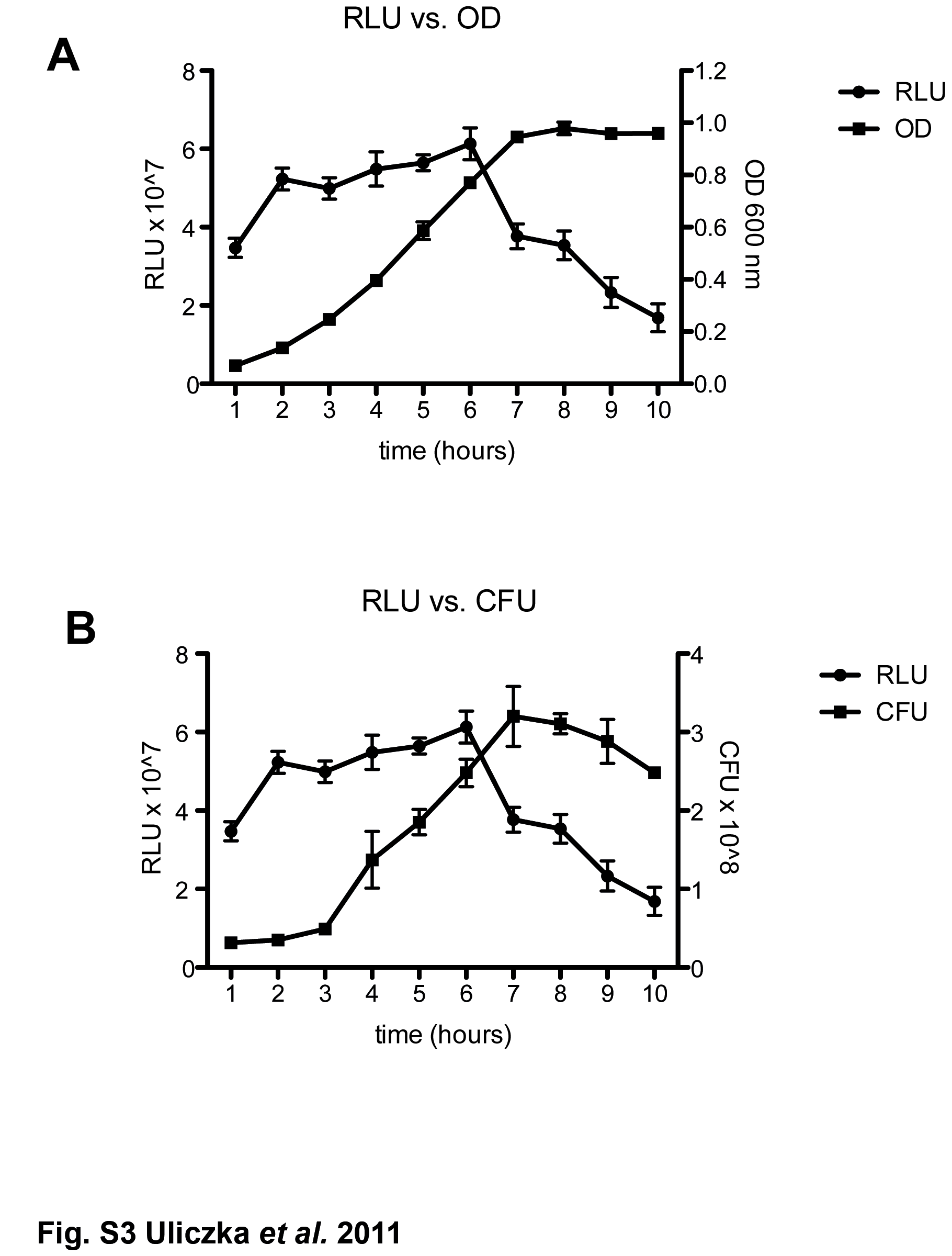

Supplement: Figure S3 — Analysis of gapA-luxCDABE expression along the bacterial growth curve. Y. pseudotuberculosis YPIII pFU166 was diluted 1∶100 grown in LB medium at 37°C to late stationary phase. Every hour, an aliquot of the culture was removed, luminescence (relative light units - RLU) and optical density (OD) at 600 nm was determined, and dilutions of the aliquot were plated onto LB to determine the colony forming units (CFU) in the culture at the indicated time point. The RLU versus OD is given in A, and RLU versus CFU is illustrated in B. (TIF) [file pone.0020425.s003.tif]

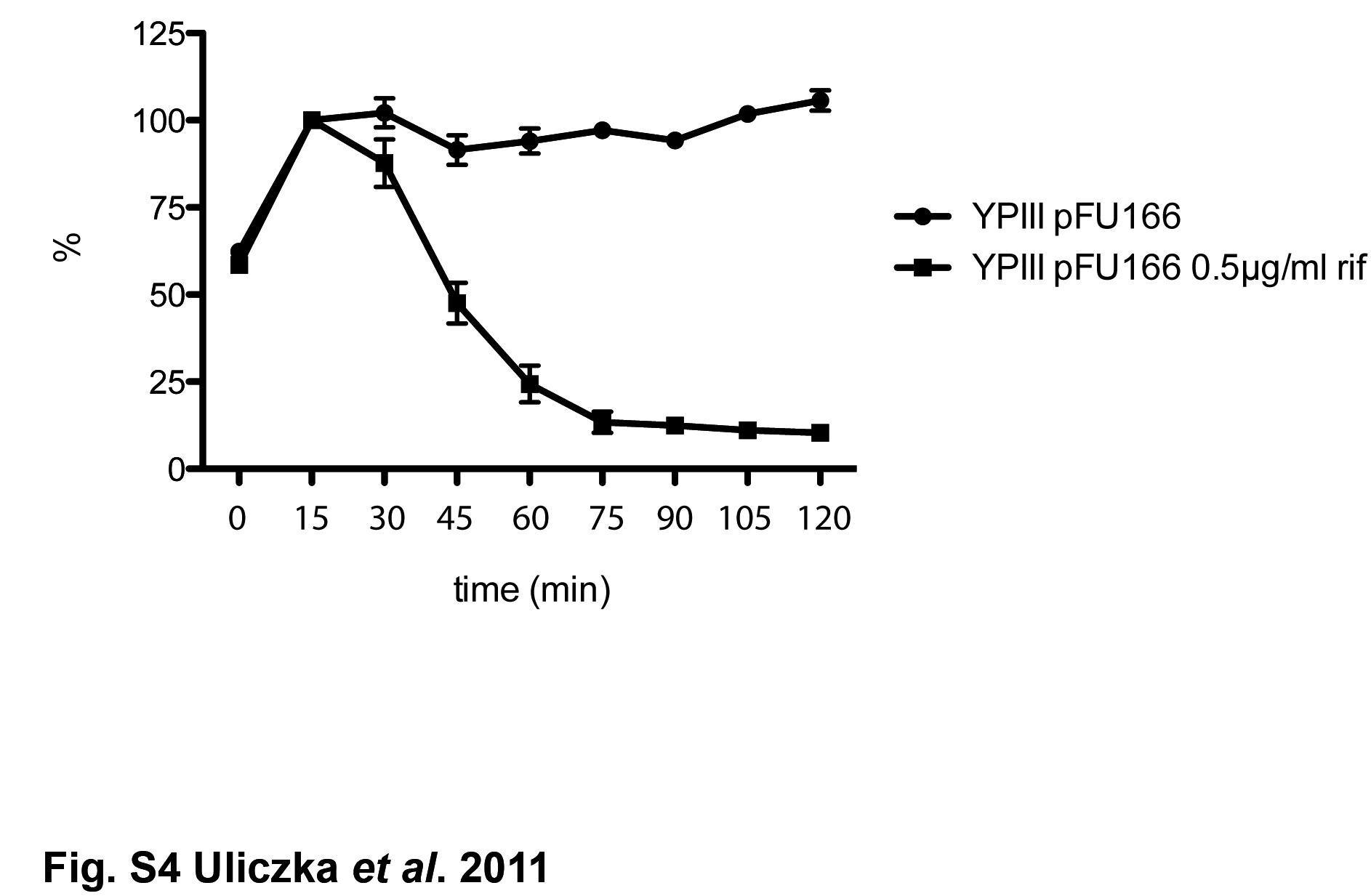

Supplement: Figure S4 — Analysis of the kinetic of gapA-luxCDABE expression. Y. pseudotuberculosis YPIII pFU166 was diluted 1∶100 in LB medium and grown at 37°C to exponential phase (OD600 = 0.6). Subsequently, rifampicin (0.5 µg/ml) was added to block transcription. Every 15 min, an aliquot of the culture was removed and luminescence (relative light units - RLU) and optical density (OD600) was determined. (TIF) [file pone.0020425.s004.tif]

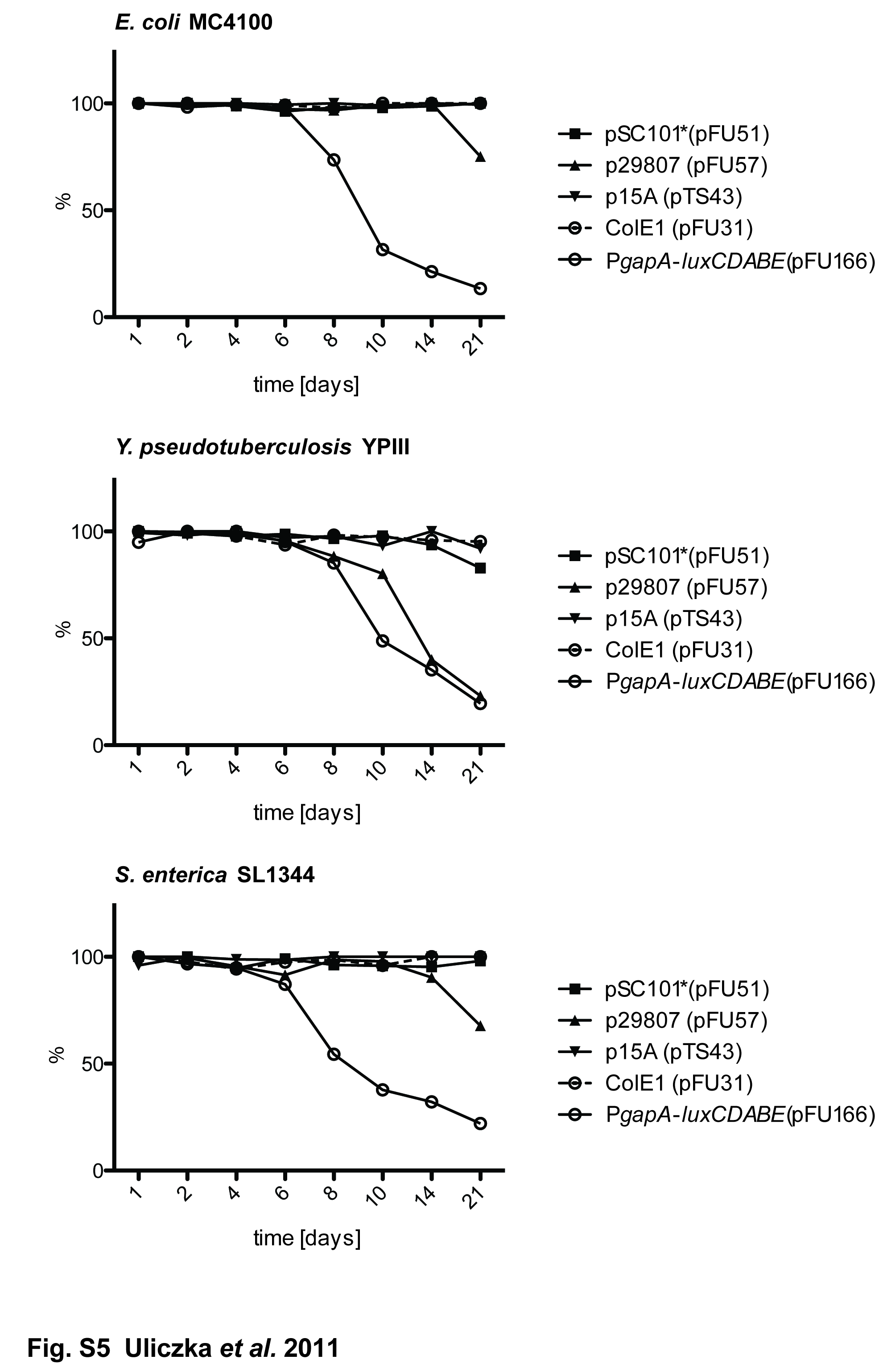

Supplement: Figure S5 — Stability of the pFU plasmids in Enterobacteriaceae without antibiotic selection. E. coli MC4100, Y. pseudotuberculosis YPIII and S. typhimurium SL3144 harboring plasmids pFU31, pFU51, pFU57, pFU166 or pTS43 were grown for 21 days in LB medium without antibiotics. Every day, 50% of the culture was inoculated with the identical volume of fresh LB medium. At indicated time points, an aliquot of the culture was removed and plated onto LB with and without antibiotics to test for the presence of the fusion plasmids. (TIF) [file pone.0020425.s005.tif]
